# Targeting CD44 and EpCAM with Antibody Dye Conjugates for the Photoimmunotherapy of Prostate Cancer

**DOI:** 10.3390/antib14010005

**Published:** 2025-01-09

**Authors:** Isis Wolf, Susanne Schultze-Seemann, Christian Gratzke, Philipp Wolf

**Affiliations:** 1 Department of Urology, Medical Center—University of Freiburg, 79106 Freiburg im Breisgau, Germany; isis.wolf@uniklinik-freiburg.de (I.W.); susanne.schultze-seemann@uniklinik-freiburg.de (S.S.-S.); christian.gratzke@uniklinik-freiburg.de (C.G.); 2 Faculty of Medicine, University of Freiburg, 79110 Freiburg im Breisgau, Germany; 3 Faculty of Biology, University of Freiburg, 79104 Freiburg im Breisgau, Germany

**Keywords:** prostate cancer, photoimmunotherapy, CD44, EpCAM, antibody dye conjugates

## Abstract

Background/Objectives: Photoimmunotherapy (PIT) is an innovative approach for the targeted therapy of cancer. In PIT, photosensitizer dyes are conjugated to tumor-specific antibodies for targeted delivery into cancer cells. Upon irradiation with visible light, the photosensitizer dye is activated and induces cancer-specific cell death. In the present article, we describe the PIT of prostate cancer (PC) as a therapeutic option for the targeted treatment of localized prostate cancer. Methods: We conjugated the silicon phthalocyanine dye WB692-CB2 to recombinant cysteine-modified anti-CD44 and anti-EpCAM antibodies via a maleimide linker and tested the antibody dye conjugates for PIT on PC cells and prostate cancer stem cell (PCSC)-like cells. Results: The anti-CD44 and anti-EpCAM antibody dye conjugates showed specific binding and high cytotoxicity against PC and PCSC-like cells following irradiation with red light. Combined treatment with both conjugates led to enhanced cytotoxic effects. Conclusions: PIT with our dye WB692-CB2 can serve as an effective focal therapy against prostate cancer, preserving the prostate gland and minimizing side effects. It can be employed during radical prostatectomy (RP) to treat residual tumor cells or lymph node metastases in areas where further surgical intervention is not feasible. Utilizing multiple conjugates against antigens expressed on differentiated PC and PCSC-like cells, such as CD44 and EpCAM, could be an effective method to eradicate residual cancer cells in heterogeneous tumors. This approach could reduce the risk of local recurrence after RP and thus increase the therapeutic outcome of PC patients.

## 1. Introduction

Prostate cancer (PC) is the second most common cancer in men from industrial countries with over 1.46 million new cases and 396,000 deaths reported annually [1]. Demographic changes and increasing life expectancy indicate that annual new PC cases will increase to 2.9 million in 2040 [2].

Treatment options for localized PC include radical prostatectomy (RP), active surveillance, watchful waiting, external beam radiation and brachytherapy [3]. The prerequisite for a successful treatment is the complete eradication of the tumor. In the case that residual tumor cells persist in the surgical margins, the tumor may recur locally and begin to metastasize. Biochemical recurrence affects approximately 20–40% of patients within ten years after RP [4]. Androgen deprivation therapy (ADT), either as monotherapy or in combination with radiotherapy and/or chemotherapy, has become the standard treatment for patients with relapsed castration-sensitive PC [3]. Unfortunately, the efficacy of ADT is limited. Over time, the tumor becomes hormone refractory and progresses to metastatic castration-resistant PC (mCRPC). The standard treatment for mCRPC involves ADT in combination with chemotherapy and prednisolone. However, this approach is primarily palliative and only marginally prolongs the patient’s overall survival, which is on average 16 to 18 months [5]. Depending on the treatment and the progression of the disease, patients suffer from a range of symptoms and adverse effects such as incontinence, erectile dysfunction, decreased libido, reduction in muscle mass and bone density, edema and psychosomatic symptoms, which negatively affect their quality of life [3]. Given the limitations of current therapeutic strategies, there is a need for the development of novel and innovative therapeutic approaches for PC.

Despite the promising results of conventional and PSMA-targeted therapies, tumor heterogeneity remains a major cause of treatment failure in PC [6]. Patients with primary PC often harbor multiple distinct tumor foci within the prostate gland, exhibiting high levels of intra-tumoral heterogeneity [7]. Prostate tumor heterogeneity refers to cancer cells with differences in morphology, transcriptional profiles, metabolism and metastatic potential. Factors that contribute to tumor heterogeneity are genetic mutations, epigenetic alterations, influence of the tumor microenvironment, evolutionary pressure and tumor cell plasticity [7,8,9]. Tumor heterogeneity can also be explained by the existence of the prostate cancer stem cell (PCSC) hypothesis, which postulates that PCSCs form a small subset of cells within the tumor capable of self-renewal and differentiate into various cell types. From a therapeutic point of view, this means that all tumor cells, i.e., PCSCs and differentiated PC cells, have to be eliminated to achieve a cure. Since PCSCs are characterized by unique tumor antigens, either exclusive to them or shared with differentiated PC cells, they can be targeted using RNA knockdown, inhibitors or antibodies directed against these antigens [10].

In the present study, we introduce photoimmunotherapy (PIT) as an innovative supportive treatment during RP to target PC cells and PCSC-like cells. PIT employs tumor-specific antibodies conjugated with photoactivatable dyes (photosensitizers) that can be activated by visible light to selectively induce cancer cell death [11]. In our approach, we used antibodies against the cluster of differentiation 44 antigen (CD44) and the epithelial cell adhesion molecule (EpCAM) to target both PC cells and PCSC-like cells. CD44 is a hyaluronic acid transmembrane receptor, which serves as a cell adhesion and signaling molecule. It is associated with the self-renewal, proliferation, differentiation, invasion and metastasis of PC. EpCAM is a transmembrane glycoprotein with various cellular functions, contributing to cancer stem cell maintenance, proliferation, invasion, metastasis and therapeutic resistance [10]. We conjugated the anti-CD44 and anti-EpCAM antibodies to our silicon phthalocyanine photosensitizer dye, WB692-CB2, and evaluated these antibody dye conjugates for PIT of PC and PCSC-like cells.

## 2. Materials and Methods

### 2.1. Cell Lines and Dye

The suspension cell line EXPI293F (Thermo Fisher Scientific, Waltham, MA, USA) was propagated in serum-free FreeStyle F17 medium (Thermo Fisher Scientific) with 8 mM L-glutamine (PAN-Biotech, Aidenbach, Germany) and 0.1% pluronic F68 (PAN-Biotech). The PC cell line PC3-PSMA was a kind gift from P. Giangrande (University of Iowa, Iowa City, IA, USA). The cell line PC3-MM2 was kindly provided by the MD Anderson Cancer Center (Huston, TY, USA). This cell line is marked by different stem cell characteristics, e.g., expression of the stem cell markers CD44, CD133, ß1 integrin or ALDH1, sphere formation, high tumorigenic and metastatic potential, and therapeutic resistance, and can therefore serve as a PCSC-like cell line [12,13,14]. Both cell lines were cultivated in RMPI 1640 medium supplemented with 10% fetal bovine serum (FBS, Merck, Darmstadt, Germany) and 1% penicillin/streptomycin (Pen/Strep, Merck). The control cell line CHO (Thermo Fisher Scientific) was cultivated in Ham’s F-12 Nutrient Mixture medium (Thermo Fisher Scientific) with 10% FBS and 1% Pen/Strep. All cell lines were propagated under sterile conditions at 37 °C in a 5% CO_2_ atmosphere. Cell line identities were confirmed using short tandem repeat (STR) analysis (CLS GmbH, Eppelheim, Germany). Lysates were generated through lysis of cells in 50 mM Tris-HCl, 150 mM NaCl, 1 mM EDTA, 0.5% sodium deoxycholate, 0.05% SDS, 1% Igepal supplemented with cOmplete^TM^ Protease Inhibitor Cocktail for 30 min on ice, and centrifugation at 18,000× *g* and 4 °C for 30 min. Protein concentration of the supernatant was determined by using the Quick Start^TM^ Bradford Protein Assay according to manufacturer’s instructions (Bio-Rad Laboratories Inc., Hercules, CA, USA). The phthalocyanine photosensitizer dye WB692-CB2 [15] was kindly provided by Jonas Storz and Reinhard Brückner, Institute for Organic Chemistry, University of Freiburg, Germany.

### 2.2. Cloning, Expression and Purification of the Anti-CD44 and Anti-EpCAM Antibodies

The genes encoding the human IgG1 heavy chains of the anti-CD44 antibody [16] and the anti-EpCAM antibody [17] each containing the cysteine mutations T120C and D265C (EU numbering) were synthesized (GeneArt, Invitrogen, Regensburg, Germany) and cloned into the expression vector pCSEH1c [18]. In our previous studies, both mutations were shown not to influence the specificity of an antibody targeting the prostate specific antigen by protein modeling [15]. Synthesized variable domains of the light chains (V_L_) of both antibodies were cloned into the expression vector pCSL3k, containing a constant domain (CL) of a human IgG1 kappa light chain. Vectors were transformed into XL1-Blue MRF’ supercompetent *E. coli* cells (Agilent Technologies, Waldbronn, Germany) and purified with help of the NucleoBond^®^ Xtra Maxi Kit (Macherey-Nagel, Düren, Germany). The sequences of the antibody chains were then verified for accuracy through sequencing (Microsynth Seqlab, Göttingen, Germany). Antibody expression in EXPI293F cells was performed as published previously [15]. After purification by protein G affinity chromatography (Cytiva, Marlborough, USA) and dialysis against PBS, concentrations of the antibodies, called CD44^T120C/D265C^ and EpCAM^T120C/D265C^, were determined by Pierce BCA Protein Assay Kit (Thermo Fisher Scientific).

### 2.3. Conjugation of the Photosensitizer Dye WB692-CB2

For dye conjugation, 1 mg of the cysteine-modified antibodies CD44^T120C/D265C^ or EpCAM^T120C/D265C^ were diluted in PBS containing 1 mM EDTA (pH 7.4) and were reduced using 40 molar equivalents of Tris-(2-Carboxyethyl)phosphine Hydrochloride (TCEP, Carl Roth, Karlsruhe, Germany) at 37 °C for 3 h. The antibodies were then dialyzed overnight at 4 °C against PBS containing 1 mM EDTA (pH 7.4). Subsequently, re-oxidation was performed with 30 molar equivalents of dehydroascorbic acid (dHAA, Sigma-Aldrich, St. Louis, MI, USA) at room temperature for 4 h. For conjugation, the antibodies were incubated with 10 molar equivalents of WB692-CB2 at room temperature for 1 h. The reaction was quenched by adding 25 molar equivalents of N-acetyl-L-cysteine (Sigma-Aldrich) for 15 min, followed by overnight dialysis against PBS (pH 7.4) [15]. Unbound dye was removed by protein G affinity chromatography and the antibody dye conjugates were stored a 4 °C protected from light. The dye-to-antibody ratios of the conjugates were determined as published [15].

### 2.4. SDS-PAGE and Western Blots

Cell lysates, antibodies and conjugates were analyzed by SDS-PAGE using the NuPAGE^®^ Bis-Tris Electrophoresis System (Thermo Fisher Scientific). For SDS-PAGE under reducing conditions, 3 µg of antibody or conjugate was combined with NuPAGE^®^ LDS Sample Buffer and NuPAGE^®^ Reducing Agent, followed by incubation for 10 min at 70 °C. Then, the samples were loaded onto a pre-cast NuPAGE^®^ Novex^®^ Bis-Tris Gel (Thermo Fisher Scientific). Electrophoresis was performed at 200 V for 35 min using the XCell SureLockTM Mini-Cell system (Thermo Fisher Scientific), with NuPAGE^®^ MES Buffer supplemented with NuPAGE^®^ Antioxidant as running buffer. For SDS-PAGE under non-reducing conditions, the same protocol using Laemmli sample buffer without NuPAGE^®^ Reducing Agent was used. Protein detection was achieved by Coomassie staining or fluorescence-based imaging (λ_max_ = 680 nm, IVIS Spectrum, In Vivo Imaging System, PerkinElmer, Waltham, MA, USA).

For Western blot analysis, the proteins were blotted onto nitrocellulose membranes at 30 V for 1 h in NuPAGE^®^ Transfer Buffer supplemented with 10% methanol and 1% NuPAGE^®^ antioxidant. The membrane was blocked with 5% non-fat milk in 0.1% Tween-20/PBS (M-PBST) for 1 h, followed by incubation with mouse anti-human CD44 (#ab82529, Abcam, Cambridge, UK) or mouse anti-human EpCAM (#sc-25308, Santa Cruz Biotechnology, Dallas, TX, USA) as primary antibodies overnight at 4 °C in M-PBST. After washing with 0.1% Tween-20/PBS, the membrane was incubated with the horseradish peroxidase (HRP)-conjugated secondary antibody rabbit anti-mouse Ig/HRP (#P0161, DAKO, Hamburg, Germany) for 1 h, followed by another washing step. ß-actin as loading control was detected using the antibody mouse α-human β-actin-HRP (Proteintech Group Inc., Rosemont, IL, USA). Detection of the target proteins was conducted using an enhanced chemiluminescence solution and the INTAS Chemo Star Imager (INTAS Science Imaging Instruments, Göttingen, Germany).

### 2.5. Flow Cytometry

Specific binding of the antibodies or antibody dye conjugates to the PC cells was evaluated by flow cytometry. An amount of 2 × 10^5^ cells per well was seeded in flow cytometry buffer consisting of PBS, 3% FBS, 0.1% sodium azide. Cells were then incubated with different concentrations of antibody or conjugate for one hour on ice. Following this, the cells were washed with PBS and incubated using a goat-anti-human Ig(H+L)-RPE detection antibody (Southern Biotech, Birmingham, AL, USA) for 30 min at 4 °C. After washing, cells were resuspended in flow cytometry buffer supplemented with 1 µg/mL propidium iodide. Mean fluorescence intensities of the stained cells were measured with a FACS Calibur Flow Cytometer and the CellQuest Pro software 4.0.2 (BD Biosciences, Heidelberg, Germany). Binding affinities of the antibodies or conjugates were determined using GraphPad Prism software 8.01. Dissociation constants (K_d_) were determined by identifying the concentration of the antibody or conjugate required to achieve half-maximal specific binding.

### 2.6. Photoimmunotherapy

For PIT, 2.5 × 10^5^ cells were seeded in 35 mm cell culture dishes and cultivated overnight at 37 °C, 5% CO_2_. On the next day, fresh medium containing the appropriate treatment (medium, free dye, 10 µg/mL antibody, 10 µg/mL conjugate or 10 µg/mL of two conjugates each in the combination experiment) was administered to the cells, followed by incubation for 24 h. After washing with PBS, cells were incubated in fresh medium and irradiated with various mean irradiation light doses (λ_max_ = 690 nm) of a light-emitting diode (LED, L690-66-60, Marubeni America Corporation, Santa Clara, CA, USA). At 24 h after irradiation, cells were trypsinized and stained with Erythrosine B (Logos, Biosystems, Anyang-si, South Korea), followed by cell viability analysis with a Neubauer Counting Chamber. Numbers of living cells were normalized relative to the untreated control.

## 3. Results

**Figure 1:**
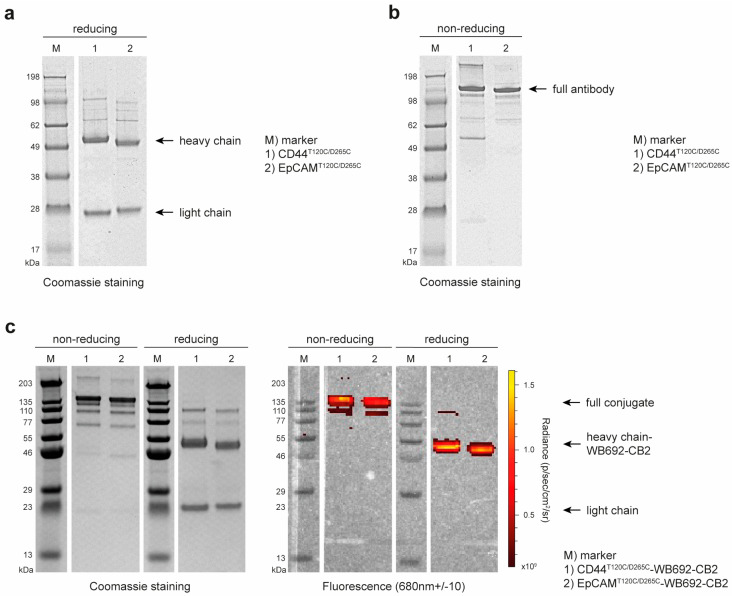
Generation of the anti-CD44 and anti-EpCAM antibody dye conjugates. (**a**) SDS gel of the purified antibodies CD44^T120C/D265C^ and EpCAM^T120C/D265C^ under reducing conditions; (**b**) SDS gel of the purified antibodies CD44^T120C/D265C^ and EpCAM^T120C/D265C^ under non-reducing conditions; (**c**) SDS gels of the purified antibody dye conjugates CD44^T120C/D265C^-WB692-CB2 and EpCAM^T120C/D265C^-WB692-CB2 under reducing and non-reducing conditions, and under white and red light.

The recombinant cysteine-engineered antibodies, CD44^T120C/D265C^ and EpCAM^T120C/D265C^, were successfully cloned and expressed in EXPI293F cells. Antibody yields were obtained, with 44.6 mg/L supernatant for CD44^T120C/D265C^ and of 39.4 mg/L supernatant for EpCAM^T120C/D265C^, in high purity after Protein G affinity chromatography (Figure 1a). SDS-PAGE under non-reducing conditions confirmed the self-assembly of the antibodies with two heavy and two light chains each, forming a whole hIgG1 molecule with a predicted size of ~150 kDa (Figure 1b).

Following dye conjugation, the antibody dye conjugates CD44^T120C/D265C^-WB692-CB2 and EpCAM^T120C/D265C^-WB692-CB2 were also analyzed by SDS-PAGE. Examination of the gels under red light (λ_max_ = 680 nm) confirmed the successful conjugation of the dye to the heavy chains of the antibodies (Figure 1c). Mean dye-to-antibody ratios were determined to be 2.82 ± 0.02 for the CD44^T120C/D265C^-WB692-CB2 conjugate and 3.14 ± 0.17 for the EpCAM^T120C/D265C^-WB692-CB2 conjugate.

**Figure 2:**
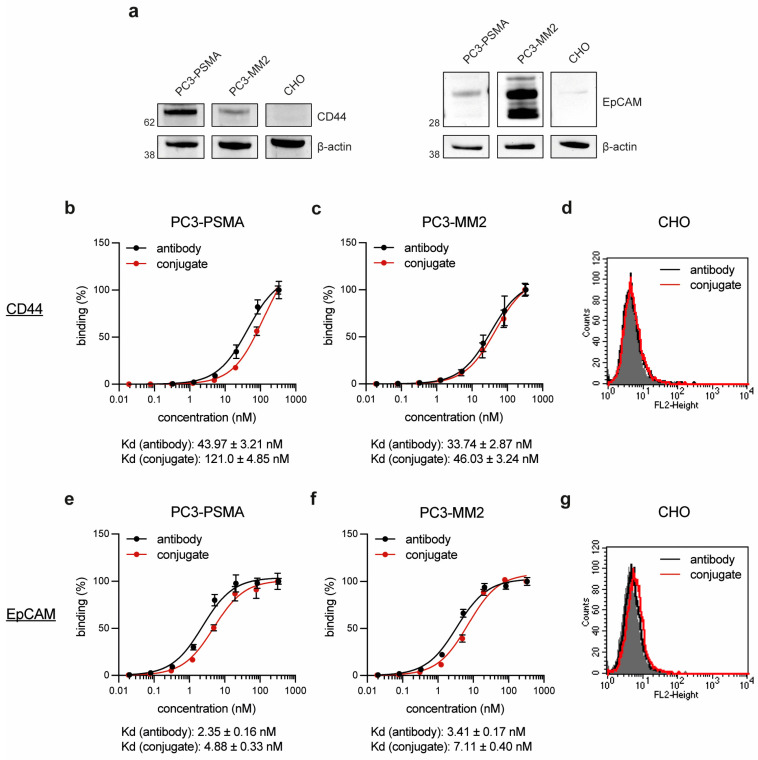
Cell binding of the antibodies and antibody dye conjugates. (**a**) CD44 and EpCAM expression of the target cells. Concentration-dependent binding of the antibody CD44^T120C/D265C^ and the conjugate CD44^T120C/D265C^-WB692-CB2 to (**b**) PC3-PSMA and (**c**) PC3-MM2 cells; (**d**) binding to CD44 negative CHO cells at a saturation concentration of 20.8 nM. Concentration-dependent binding of the antibody EpCAM^T120C/D265C^ and the conjugate EpCAM^T120C/D265C^-WB692-CB2 to (**e**) PC3-PSMA and (**f**) PC3-MM2 cells; (**g**) binding to EpCAM negative CHO cells at a saturation concentration of 20.8 nM. Mean values ± SD of three independent experiments. Abbreviation: Kd, dissociation constant.

In the subsequent step, Western Blot analyses confirmed the expression of the target proteins CD44 and EpCAM in the prostate cancer cell line PC3-PSMA and the prostate cancer stem cell-like (PCSC-like) cell line PC3-MM2, whereas the control cell line CHO showed no expression of either antigen (Figure 2a). The binding affinity of the conjugates CD44^T120C/D265C^-WB692-CB2 and EpCAM^T120C/D265C^-WB692-CB2 were then evaluated on both cell lines. The conjugates exhibited slightly lower binding affinities (higher Kd values) within the low nanomolar range compared to their uncoupled counterparts, CD44^T120C/D265C^ and EpCAM^T120C/D265C^ (Figure 2b,c,e,f). No binding was detected with both molecules on CD44 and EpCAM-negative CHO cells (Figure 2d,g). This proved that the dye conjugation did not affect or only minimally affected the avidity of the antibodies and did not result in unspecific binding of the conjugates.

**Figure 3:**
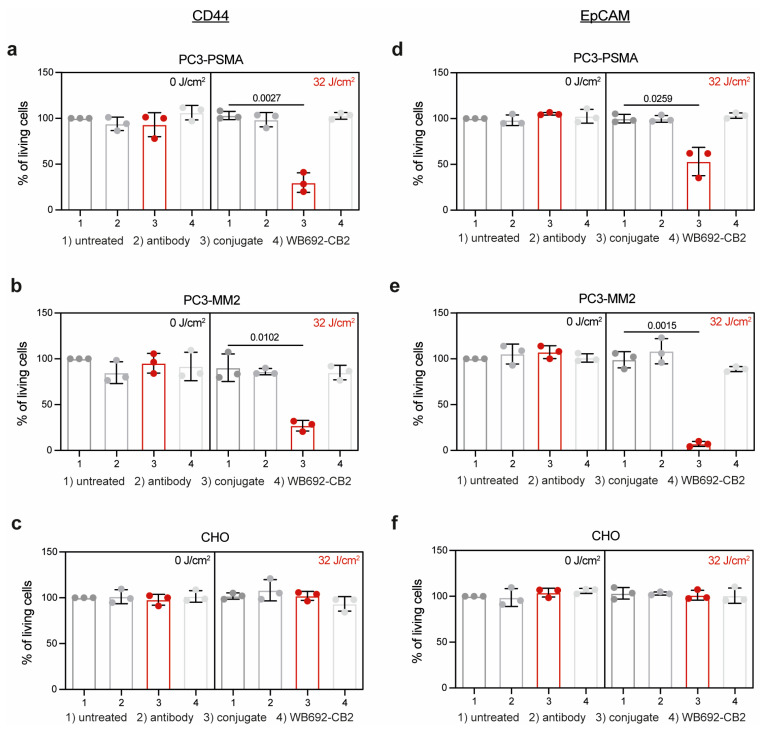
PIT of PC or PCSC-like cells with the conjugates CD44^T120C/D265C^-WB692-CB2 and EpCAM^T120C/D265C^-WB692-CB2. (**a**) Viability of PC3-PSMA, (**b**) PC3-MM2 or (**c**) CHO cells 24 h after PIT incubated with 10 µg/mL CD44^T120C/D265C^, 10 µg/mL CD44^T120C/D265C^-WB692-CB2, free dye or medium (untreated control), and irradiation with a light dose of 32 J/cm^2^. Control cells were not irradiated (0 J/cm^2^). Viability of (**d**) PC3-PSMA, (**e**) PC3-MM2 and (**f**) CHO cells 24 h after PIT incubated with 10 µg/mL EpCAM^T120C/D265C^, 10 µg/mL EpCAM^T120C/D265C^-WB692-CB2, free dye or medium (untreated control), and irradiated with a light dose of 32 J/cm^2^. Control cells were not irradiated (0 J/cm^2^). Mean values ± SD of three independent biological experiments. Statistical analyses by Student’s *t*-test (unpaired, parametric with Welch’s correction).

The efficacy of PIT using the conjugates CD44^T120C/D265C^-WB692-CB2 and EpCAM^T120C/D265C^-WB692-CB2 was evaluated on the target cells 24 h after exposure with a light dose of 32 J/cm^2^. Control cells remained untreated or were incubated with equimolar concentrations of antibody or free dye. The viability of PC3-PSMA and PC3-MM2 cells treated with the conjugate CD44^T120C/D265C^-WB692-CB2 were significantly reduced to 29.9 ± 10.7% (*p* = 0.0027) and 27.1 ± 5.8% (*p* = 0.0102), respectively (Figure 3a,b). In contrast, no cytotoxic effects were observed in cells treated with the antibody or free dye. Cells from all treatment groups remained alive without irradiation. Moreover, CD44-negative CHO cells were completely unaffected by PIT (Figure 3c). Similar results were obtained with the conjugate EpCAM^T120C/D265C^-WB692-CB2 in PC3-PSMA and PC3-MM2 cells after PIT. Cell viability was significantly reduced to 53.1 ± 15.4% (*p* = 0.0259; Figure 3d) and 7.1 ± 2.8% (*p* = 0.0015; Figure 3e), respectively, while EpCAM-negative CHO cells were not impaired by treatment (Figure 3f). These findings indicate that PIT using our dye WB692-CB2 is a highly selective and effective therapeutic strategy for targeting PC cells and PCSCs.

**Figure 4:**
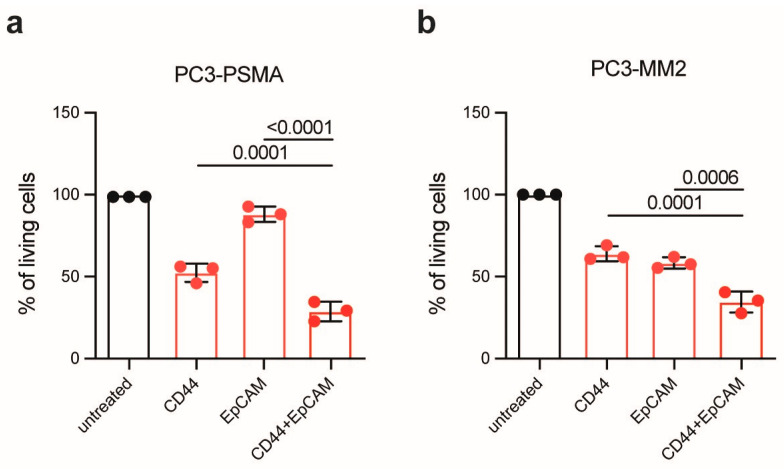
Combined PIT of PC cells and PCSC-like cells. (**a**) Viability of PC3-PSMA cells and (**b**) PC3-MM2 cells 24 h after PIT with 10 µg/mL CD44^T120C/D265C^-WB692-CB2 or EpCAM^T120C/D265C^-WB692-CB2 alone or in combination, and a light dose of 16 J/cm^2^. Mean values ± SD of three independent biological experiments. Statistical analyses were performed using one-way ANOVA, followed by Tukey’s multiple comparison test for post hoc analysis, with an adjusted significance level of *p* = 0.05.

To develop a therapeutic approach against residual PC cells following RP, the efficacy of PIT using CD44^T120C/D265C^-WB692-CB2 in combination with EpCAM^T120C/D265C^-WB692-CB2 was evaluated using a reduced light irradiation dose of 16 J/cm^2^. In the PC cell line PC3-PSMA, combinatorial PIT led to a significant reduction in cell viability compared to single treatments. Specifically, cell viability was significantly reduced from 52.5 ± 5.6% targeting CD44 and 88.1 ± 4.7% targeting EpCAM to only 28.9 ± 6.0% after PIT targeting CD44 in combination with EpCAM (*p* = 0.0001 and *p* < 0.0001, respectively; Figure 4a). Similar results were obtained in the PCSC-like PC3-MM2 cells, where the cell viability was significantly reduced from 64.0 ± 4.5% targeting CD44 and 58.4 ± 3.4% targeting EpCAM to 34.6 ± 6.5% after combinatorial PIT targeting CD44 and EpCAM (*p* = 0.0001 and *p* = 0.0006, respectively; Figure 4b).

Our results demonstrated the enhanced efficacy of combined PIT compared to single PIT, indicating that simultaneously targeting multiple tumor antigens may provide a more effective therapeutic strategy for PC.

## 4. Discussion

Tumor heterogeneity in PC remains a major challenge, contributing to therapeutic resistance and tumor recurrence. Consequently, there is an urgent need for the development of novel and innovative therapeutic approaches for PC. PIT has emerged as a new cornerstone in local cancer treatment with minimal off-target effects. This technique leverages photoactivatable photosensitizers conjugated to antibodies or antibody fragments to induce specific, immunogenic cancer cell death and systemic antitumor immunity [19]. We have developed the silicon phthalocyanine dye, WB692-CB2, which can be directly conjugated to free cysteines in antibodies via a maleimide linker [15]. Since all naturally occurring cysteines in an antibody molecule are used for the formation of intra- and interchain disulfide bridges, our dye can be specifically conjugated to engineered cysteines, which are not expected to affect the folding, and thus the avidity and specificity of the antibody. The cysteine-specific conjugation offers an advantage over the conventional coupling to lysines. A human IgG1 antibody contains approx. 80 to 90 lysines, leading to undefined and heterogeneous conjugate preparations, when coupling occurs at these sites. By specifically targeting engineered cysteines, we can achieve more homogenous antibody dye conjugate preparations. Our conjugation method allowed the attachment of approximately 2.8 to 3.1 dye molecules to the four engineered cysteines of one each of anti-CD44 and anti-EpCAM antibody molecules.

Our PIT experiments demonstrated that the anti-CD44 and anti-EpCAM conjugates, with their specific dye loads, effectively induced the cell death of both PC and PCSC-like cells within 24 h after irradiation using a light dose of 32 J/cm^2^. Antigen-negative control cells were not affected by the treatment, which demonstrates the high selectivity of our approach. Despite differences in CD44 expression levels, the therapeutic effects of PIT targeting CD44 were similar in PC3-MM2 and PC3-PSMA cells. This discrepancy can be explained by the fact that the Western Blot data reflect total CD44 expression levels in the cells, but do not distinguish between intracellular and surface expression. Since PIT efficacy depends on the binding of the conjugates to surface antigens, it is possible that surface-accessible CD44 levels are more comparable between these cell lines than the total expression levels would suggest. Furthermore, intrinsic differences in susceptibility to PIT-induced cytotoxicity may also contribute to the equivalent therapeutic outcomes observed.

Combined PIT using both conjugates significantly enhanced cytotoxicity compared to single treatments in an additive manner. This could substantially enhance therapeutic outcomes compared to single treatments in the clinic. In addition to the combination of different conjugates against different target antigens, it is also possible to achieve better effects by increasing the light dose [15]. Moreover, engineering antibodies to incorporate additional cysteines could increase dye loading in future applications, which could also lead to an increased cytotoxicity. Further in vitro and in vivo studies are needed to validate the proof of principle for PIT using anti-CD44 and anti-EpCAM antibodies against PC, and to assess its potential as an effective option for future clinical use.

The un-conjugated dye exhibited no cytotoxicity, likely due to its inherent hydrophilicity, which enhances solubility and inhibits its diffusion across cellular membranes [15]. This characteristic is particularly advantageous, as it ensures that the dye exhibits cytotoxic effects only, when it is transported by the antibody into the target cells and is activated by light. This might reduce potential side effects in clinical practice and enhances the specificity of the therapeutic approach. Recently, we found that a main mechanism of PIT-induced cell death with our dye is pyroptosis [15]. Pyroptosis is a distinct type of pro-inflammatory programmed cell death, primarily driven by caspase-1 dependent cleavage of gasdermin D. This cleavage leads to the formation of non-selective pores in the plasma membrane, allowing the release of pro-inflammatory cytokines and influx of water. This is followed by cellular swelling, membrane rupture and release of damage-associated molecular patterns and tumor-associated neoantigens, which initiate an adaptive anti-cancer immune response [20]. Thus, locally applied PIT could potentially lead to systemic immunological antitumor effects. This means that, in clinical use, intraoperative PIT could also be effective against tumor cells that have already spread or metastasized. To further explore this, future studies can employ syngeneic tumor models in immunocompetent mice to investigate the immunogenic effects of PIT [21].

## 5. Conclusions

In the context of PC, WB692-CB2-based PIT can serve as a focal treatment approach, allowing for preservation of the prostate gland and minimizing side effects. It can be utilized during minimally invasive robotic-assisted RP to detect and treat residual tumor cells or lymph node metastases in areas where surgery cannot be continued in order to protect adjacent structures like the rectum or nerves responsible for urinary and sexual function. In clinical use, activation of the antibody dye conjugates during intraoperative PIT will be influenced by two main factors: firstly, by light accessibility of the tumor and, secondly, by tissue penetration of the conjugates. Light with a wavelength of around 700 nm, which can activate our dye, shows a maximum penetration depth of several millimeters in tissue, since it is only minimally absorbed by water or hemoglobin [22]. Light sources for illuminating the antibody dye conjugates could be easily implemented in robot surgical systems for RP in the future. The tissue penetration of the antibody dye conjugates depends on the antigen density, the tumor microenvironment, and the density and morphology of the tumor vessels [23]. Since the application of PIT in our case is limited to the outer margins after removal of the prostate, rather than deeper tumor areas, there is a chance that enough antibody dye conjugates have accumulated there to enable effective PIT. The use of multiple conjugates against antigens expressed on differentiated PC cells and PCSC cells, e.g., CD44 and EpCAM, could be an effective method to eradicate the heterogeneous population of residual cancer cells completely. This approach could lead to the prevention of local recurrence after RP and thus increase the therapeutic outcome of PC patients.

## Data Availability

The raw data supporting the conclusions of this article will be made available by the authors on request.

## References

[1] Bray F., Laversanne M., Sung H., Ferlay J., Siegel R.L., Soerjomataram I., Jemal A. (2024). Global cancer statistics 2022: Globocan estimates of incidence and mortality worldwide for 36 cancers in 185 countries. CA Cancer J. Clin..

[2] James N.D., Tannock I., N’Dow J., Feng F., Gillessen S., Ali S.A., Trujillo B., Al-Lazikani B., Attard G., Bray F. (2024). The lancet commission on prostate cancer: Planning for the surge in cases. Lancet.

[3] Rebello R.J., Oing C., Knudsen K.E., Loeb S., Johnson D.C., Reiter R.E., Gillessen S., Van der Kwast T., Bristow R.G. (2021). Prostate cancer. Nat. Rev. Dis. Primers.

[4] Ward J.F., Moul J.W. (2005). Rising prostate-specific antigen after primary prostate cancer therapy. Nat. Clin. Pract. Urol..

[5] Karantanos T., Corn P.G., Thompson T.C. (2013). Prostate cancer progression after androgen deprivation therapy: Mechanisms of castrate resistance and novel therapeutic approaches. Oncogene.

[6] Ge R., Wang Z., Cheng L. (2022). Tumor microenvironment heterogeneity an important mediator of prostate cancer progression and therapeutic resistance. NPJ Precis. Oncol..

[7] Haffner M.C., Zwart W., Roudier M.P., True L.D., Nelson W.G., Epstein J.I., De Marzo A.M., Nelson P.S., Yegnasubramanian S. (2021). Genomic and phenotypic heterogeneity in prostate cancer. Nat. Rev. Urol..

[8] Marusyk A., Polyak K. (2010). Tumor heterogeneity: Causes and consequences. Biochim. Biophys. Acta.

[9] Proietto M., Crippa M., Damiani C., Pasquale V., Sacco E., Vanoni M., Gilardi M. (2023). Tumor heterogeneity: Preclinical models, emerging technologies, and future applications. Front. Oncol..

[10] Wolf I., Gratzke C., Wolf P. (2022). Prostate cancer stem cells: Clinical aspects and targeted therapies. Front. Oncol..

[11] Mohiuddin T.M., Zhang C., Sheng W., Al-Rawe M., Zeppernick F., Meinhold-Heerlein I., Hussain A.F. (2023). Near infrared photoimmunotherapy: A review of recent progress and their target molecules for cancer therapy. Int. J. Mol. Sci..

[12] Rowehl R.A., Crawford H., Dufour A., Ju J., Botchkina G.I. (2008). Genomic analysis of prostate cancer stem cells isolated from a highly metastatic cell line. Cancer Genom. Proteom..

[13] Lee Y.C., Jin J.K., Cheng C.J., Huang C.F., Song J.H., Huang M., Brown W.S., Zhang S., Yu-Lee L.Y., Yeh E.T. (2013). Targeting constitutively activated β1 integrins inhibits prostate cancer metastasis. Mol. Cancer Res. MCR.

[14] Botchkina G.I., Zuniga E.S., Rowehl R.H., Park R., Bhalla R., Bialkowska A.B., Johnson F., Golub L.M., Zhang Y., Ojima I. (2013). Prostate cancer stem cell-targeted efficacy of a new-generation taxoid, sbt-1214 and novel polyenolic zinc-binding curcuminoid, cmc2.24. PLoS ONE.

[15] Wolf I., Storz J., Schultze-Seemann S., Esser P.R., Martin S.F., Lauw S., Fischer P., Peschers M., Melchinger W., Zeiser R. (2024). A new silicon phthalocyanine dye induces pyroptosis in prostate cancer cells during photoimmunotherapy. Bioact. Mater..

[16] Birzele F., Cannarile M., Feuerhake F., Fischer T., Heil F., Honold K., Nopora A., Schmitt-Graeff A., Voss E., Weigand S. (2014). Markers for Responsiveness to Anti-cd44 Antibodies. Patent.

[17] Cizeau J., Macdonald G., Premsukh A. (2008). Optimized Nucleic Acid Sequences for the Expression of vb4-845. Patent.

[18] Steinwand M., Droste P., Frenzel A., Hust M., Dübel S., Schirrmann T. (2014). The influence of antibody fragment format on phage display based affinity maturation of igg. mAbs.

[19] Hsu M.A., Okamura S.M., De Magalhaes Filho C.D., Bergeron D.M., Rodriguez A., West M., Yadav D., Heim R., Fong J.J., Garcia-Guzman M. (2023). Cancer-targeted photoimmunotherapy induces antitumor immunity and can be augmented by anti-pd-1 therapy for durable anticancer responses in an immunologically active murine tumor model. Cancer Immunol. Immunother..

[20] Hou J., Hsu J.M., Hung M.C. (2021). Molecular mechanisms and functions of pyroptosis in inflammation and antitumor immunity. Mol. Cell.

[21] Kato T., Okada R., Furusawa A., Inagaki F., Wakiyama H., Furumoto H., Okuyama S., Fukushima H., Choyke P.L., Kobayashi H. (2021). Simultaneously combined cancer cell- and ctla4-targeted nir-pit causes a synergistic treatment effect in syngeneic mouse models. Mol. Cancer Ther..

[22] Ash C., Dubec M., Donne K., Bashford T. (2017). Effect of wavelength and beam width on penetration in light-tissue interaction using computational methods. Lasers Med. Sci..

[23] Thurber G.M., Weissleder R. (2011). Quantitating antibody uptake in vivo: Conditional dependence on antigen expression levels. Mol. Imaging Biol..

